# Food Insecurity and Perceived Effects of COVID-19 on Livelihoods in Rural Sri Lanka

**DOI:** 10.1177/03795721231197249

**Published:** 2023-09-13

**Authors:** Nishmeet Singh, Samuel Scott, Neha Kumar, Gayathri Ramani, Quinn Marshall, Kate Sinclair, Saman Kalupahana, Malika Fernando, Renuka Silva, Amila Perera, Renuka Jayatissa, Deanna Olney

**Affiliations:** 1 The University of Edinburgh, Scotland, United Kingdom; 2 International Food Policy Research Institute, New Delhi, India; 3 International Food Policy Research Institute, Washington, DC, USA; 4 Johns Hopkins Bloomberg School of Public Health, Baltimore, MD, USA; 5 World Food Programme, Colombo, Sri Lanka; 6 Wayamba University, Makandura, Sri Lanka; 7 Medical Research Institute, Colombo, Sri Lanka

**Keywords:** food security, agriculture, COVID-19 pandemic, livelihoods

## Abstract

**Background:**

Little is known about how the COVID-19 pandemic has affected food security and livelihoods in Sri Lanka.

**Objective:**

This article aims to assess food insecurity, perceived effects of COVID-19, and coping mechanisms among agriculture-based households in rural Sri Lanka.

**Methods:**

We used 2 rounds of panel data from phone surveys (n = 1057 households) conducted in 5 districts. Food insecurity (30-day recall), perceived impacts of COVID-19 (6-month recall), and coping mechanisms (6-month recall) were assessed using a household questionnaire. To assess food insecurity, we used the 8-item Food Insecurity Experience Scale. We tested for differences between T1 (baseline: December 2020-February 2021) and T2 (follow-up: July 2021-September 2021) and explored the association between food insecurity and the perceived effect of COVID-19 on income using a logistic regression model.

**Results:**

Food insecurity was highly prevalent (T1: 75%, T2: 80%) but varied across districts. Most respondents were affected by COVID-19 and/or COVID-19-associated mitigation measures (T1: 84%, T2: 89%). Among affected households, commonly reported impacts included those on income (T1: 77%, T2: 76%), food costs (T1: 84%, T2: 83%), and travel (∼90% in both rounds). Agricultural activities were also adversely affected (T1: 64%, T2: 69%). About half of COVID-19-affected households reported selling livestock or assets to meet basic needs. Households whose income was impacted by COVID-19 were more likely to be food insecure (adjusted odds ratio: 2.56, *P* < .001).

**Conclusions:**

Households in rural Sri Lanka experienced food insecurity and livelihood disturbances during the COVID-19 pandemic. Additional surveys are needed to assess recovery post-COVID-19 and to understand if programs that support livelihoods have been protective.

## Abstract (plain language summary)

**Plain language title:**

Food Insecurity and Perceived Effects of COVID-19 on Livelihoods in Rural Sri Lanka

**Plain language summary:**

**Background:**

- Sustained levels of high food insecurity are associated with a range of negative health, nutrition, and well-being effects.
- The COVID-19 pandemic is expected to aggravate food insecurity and worsen the livelihood situation.
- Little is known about how the COVID-19 pandemic affected food security and livelihoods of agriculture-based households in rural Sri Lanka.

**Method:**

- This original article used household level survey data from 2 rounds of phone surveys conducted in 5 districts of Sri Lanka.
- Using a household-level questionnaire, we recorded experience of food insecurity in the last 30 days, perceived impact of COVID-19, and adopted coping mechanism in the 6 months prior to the survey.
- We reported statistical means and tested for differences between 2 survey rounds.
- We also explored association between food insecurity and the perceived effect of COVID-19 on income.

**Results:**

- Household-level food insecurity was highly prevalent during the pandemic.
- Households perceived a negative effect of the pandemic on their income and employment sources.
- Households whose income was impacted by the pandemic were more likely to be food insecure.

**Conclusion:**

- Agriculture-based households in rural Sri Lanka experienced food insecurity and livelihood disturbances during the COVID-19 pandemic.
- Additional research is needed to assess recovery post COVID-19 and to understand whether livelihood support programs have been protective.

## Introduction

The COVID-19 pandemic and its associated mitigation measures continue to have an unprecedented effect on human lives. Until December 2021, an estimated 18 million people had died from the virus worldwide, and many were still facing negative physical and mental health effects.^1^ Economic livelihoods have been damaged, with an estimated 114 million people globally losing their jobs in 2020 following COVID-19 closures, leading to a total personal income loss of US$3.7 trillion.^2^ Consequently, case studies at the household level have reported reduced income, heightened food insecurity, interruption in continued learning for children due to school closures, and a lower likelihood of securing a job, especially for women, youth, self-employed, and casual workers.^3^

The COVID-19 pandemic is expected to exacerbate food insecurity beyond estimates based on prepandemic conditions^4^ through disruptions across the food system activities: production, value chains, retail, and consumption. This impact is likely to be higher in low- and middle-income countries due to their poor structural conditions and inability to respond and recover from shocks.^5^ Evidence from several South Asian countries (Bangladesh, India, Nepal, Afghanistan, and Pakistan) suggests that COVID-19 has already hampered household-level food access through price spikes, shortages, food loss, loss of remittance income, and unemployment, especially for vulnerable groups such as low-income farmers, daily workers, and women.^4,6^

Food insecurity has been a prolonged concern for Sri Lanka even before the disruptions from COVID-19. In 2019, Sri Lanka ranked 66 of 117 countries on the annual Global Hunger Index^7^ and 66 of 113 on the Global Food Insecurity Index.^8^ Within Sri Lanka, rural households, especially paddy cultivators in the agriculture sector, are often food insecure and unable to deal with income fluctuations and climatic shocks.^9,10^ Before the COVID-19 pandemic, yield stagnation, rising food prices, poor agriculture marketing infrastructure, a large informal workforce, land fragmentation and degradation, urbanization, climate change, and food safety were drivers of food insecurity in Sri Lanka.^11^ These drivers persist, and some have likely been exacerbated by the COVID-19 pandemic and associated mitigation measures, although the extent of these changes is still unclear.

Little is known about how the food security situation changed during the pandemic in Sri Lanka. Here, using data collected during the pandemic, through phone surveys in 2020 and 2021, we report on household experiences of food insecurity and perceived effects of COVID-19 and associated mitigation measures among rural households engaged in agricultural activities in Sri Lanka. First, we assess levels and trends of food insecurity experiences. Next, we investigate perceived (self-reported) effects of COVID-19 and its associated restrictions on health, livelihoods, and food availability/access, along with coping measures. Additionally, we examine the perceived impact of COVID-19 on various sources of income and agriculture activities. Finally, we test the hypothesis that the perceived impact of COVID-19 on income is positively associated with food insecurity.

## Methods

### Study Context

Sri Lanka is an island country with a population of 21.8 million, 77% of whom live in rural areas.^12^ The country is administratively structured as 9 provinces, divided into 25 districts, subdivided into 335 divisional secretariat divisions (DSDs), further split as 14 020 Grama Niladhari divisions (GNs), the smallest administrative unit.^13^ The agriculture sector employs approximately 30% of the rural working population, which includes self-farming and farm-wage labor, and the primary economic activities are paddy (rice) cultivation, fishing, and livestock rearing.^14^ Paddy is Sri Lanka’s major food crop, with cropping limited to 2 primary seasons—Dry or “Yala” (May to August) and Wet or “Maha” (September to March)—with varying paddy cultivation by season and district. For example, in the 2016 to 2017 agriculture season, paddy cultivation was done by 22% and 14% of farmers in the Maha and Yala seasons, respectively. During the Maha season of 2016 to 2017, 15% of farmers were cultivating tea, 13% vegetables, and 8% coconut; the district-level paddy cultivation varied from 4% to 60% of farmers.^15^ Common problems faced by agriculture households in Sri Lanka include climatic hazards (irregular rains/droughts), lack of finance and storage infrastructure, and low produce prices.^15^ In the last 2 decades, droughts have been severe in the dry zones of Sri Lanka (Northern, Eastern, North Western provinces, Hambantota, and Anuradhapura districts).^15,16^ Consequently, households engaged in farming are among the poorest in rural Sri Lanka.^17^

### COVID-19 in Sri Lanka

**Figure 1.**
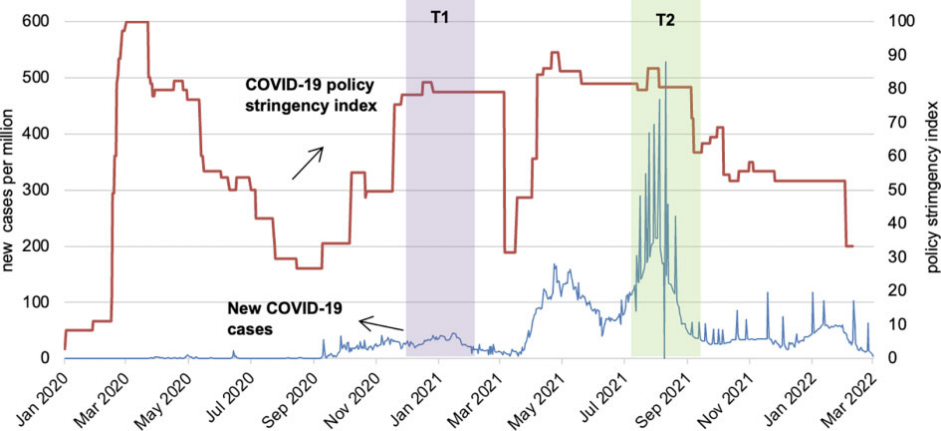
Now confirmed COVID-19 cases per million, policy stringency measures and survey rounds in Sri Lanka.

COVID-19 was first detected in Sri Lanka in February 2020, but cases were relatively low compared to other South Asian countries until October 2020. The peak of confirmed COVID-19 cases per million people on a single day between March 2020 and September 2020 was approximately 4 for Sri Lanka compared to 66 in India, 30 in Pakistan, 23 in Bangladesh, and 48 in Nepal. Between October 2020 and March 2021, there was a first peak in COVID-19 cases in Sri Lanka followed by a dip and then another wave from April to October 2021 (Figure 1).^18^ This second wave timed with the emergence of the Delta variant of SARS-CoV-2 virus that causes COVID-19, had a peak of 277 cases per million, and 9.5 deaths per million (rolling average of 7 days), the highest during the pandemic for Sri Lanka.^19^

Despite relatively low COVID-19 cases in the first few months of the pandemic from January 2020 to April 2020, the Sri Lankan government took a proactive approach to containing the spread of COVID-19 and imposed restrictions on movement of people and goods at several time points across all provinces, and full lockdowns in a few provinces.^20,21^ These measures were relaxed between May and September 2020.^22^ However, as cases increased exponentially in Sri Lanka after September 2020, an extended round of COVID-19-related lockdowns and restrictions were instituted until March 2021 and then again from May 2021 until the beginning of 2022. Figure 1 shows the number of COVID-19 cases and the variation in policy measures implemented by the Sri Lankan government between January 2020 and March 2022, using the data from the Oxford COVID-19 Policy Stringency Index (scaled 0-100).^21^

### Program Description

The data used in the analyses presented in this study were collected as part of a study designed to assess the impact of a nutrition-sensitive Food for Assets program called R5N (an acronym for the program’s focus: rural, resilience, risk reduction, reconstruction, recovery, and nutrition), implemented by the World Food Programme (WFP) in Sri Lanka. Food for Assets is a social protection program involving a cash or food transfer for work used to create or rehabilitate community assets. Food for Assets programs often include several complementary activities tailored to the context. The R5N program in Sri Lanka was designed to increase resilience of rural agricultural households through addressing agriculture production constraints. The program includes activities to improve water security through rehabilitation of community water reservoirs and improvements to households’ wells/ponds, as well as agricultural livelihoods through support to diversified agricultural income generation activities. In addition to addressing food security and resilience issues, WFP’s programs have been working to increase program impact on diet and nutrition outcomes through the inclusion of nutrition-sensitive program components.^23^ In line with this, the R5N program included health promotion activities in addition to the resilience focused activities, offered to a subset of communities, to test whether adding these activities would increase program impact on diet outcomes.

### Study Area and Design

The study occurred in 5 drought-prone districts of the country: Mullaitivu, Mannar, Batticaloa, Matale, and Monaragala (Supplemental Figure 1). In each district, the program covered 1 division (DSD). The program implementation unit was households within selected GNs. Across the 5 districts, 50 of the 117 GNs were selected by WFP and the Government of Sri Lanka to participate in the R5N program. The impact evaluation from which data for the current analysis were derived followed a quasi-experimental design to evaluate program impacts in 30 R5N GNs where implementation started in 2020 and 2021 (of which 15 were randomly selected to participate in a behavior change intervention or “Health Promotion Process”), and 15 GNs from the same DSDs were matched to the 30 R5N GNs to serve as controls. For the analyses presented in this article, we looked at changes between 2 study time points for the sample as a whole (30 R5N GNs + 15 control GNs). Ethical approval was obtained from the human subjects review boards of the International Food Policy Research Institute and Wayamba University in Sri Lanka.

### Sample Selection

For the first round of data collection, our survey team contacted all beneficiary households in the 30 R5N GNs using the beneficiary list provided by WFP **(**n = 1250, see Supplemental Table 1 for sample flow). The R5N households had to have at least 1 household member who participated in agricultural livelihood activities to participate in the program. Within the R5N households, the direct program beneficiary was selected as the survey respondent. To create our matched sample, we randomly selected nonbeneficiary households (∼1400) from the 15 control GNs using the most recent available electoral list (2016) for each GN. We aimed to oversample nonbeneficiary households to help with the matching for the impact assessment. In the control GN sample, we precontacted households and only invited those involved in agriculture activities in the past 2 years from the survey date to participate in the survey; this was done to select a control sample with potentially similar characteristics as the program sample. All the interviews were conducted with adults (≥18 years of age).

### Data Collection

Data were collected using phone surveys at T1 (baseline: December 2020-February 2021), which overlaps the first COVID wave (less severe), and T2 (follow-up: July 2021-September 2021), which overlaps the second wave (more severe, see Figure 1). The survey questionnaire was prepared in English, translated into the local languages (Sinhala and Tamil) and cross-checked, then programmed for computer-assisted telephonic interviews using SurveyCTO.^24^ Enumerators and supervisors were remotely trained in each round on the questionnaire content, conducting phone surveys using the SurveyCTO phone app and in the procedures regarding informed consent.

The T1 survey was conducted over 5 phone calls with an average call duration of 15 to 20 minutes per call. Modules included household demographics, dwelling characteristics, program exposure, food and nonfood consumption, food security, agriculture activities (including livestock), perceived COVID-19 impacts, nutrition knowledge, and dietary recall. The T2 survey was conducted with the same households that had participated in the baseline, included 3 calls, and covered a subset of baseline survey topics: household membership, program exposure, perceived COVID-19 impacts, nutrition knowledge, and dietary intake (Supplemental Table 2).

Survey participation was voluntary and confidential. During the first call, enumerators explained the research objectives, survey content, and participation risks and benefits to potential respondents. For those agreeing to participate, oral consent was obtained. A small incentive of 200 LKR (approximately US$1 during T1 and T2) of phone credit was distributed following each completed call.

### Measures

This article uses data on total household members, dependent members, sex ratio, own house and size of agriculture land holding, household assets, food insecurity, and perceived impacts of COVID-19. Food insecurity was measured using the Food and Agriculture Organisation's (FAO's) validated 8-question Food Insecurity Experience Scale (FIES), which assessed experiences at the household level in the last 30 days.^25^ The FIES questions capture gaps in food access due to lack of money or other resources across a continuum of experience from mild to severe food insecurity. Perceived COVID-19 impacts in the last 6 months were measured using self-reported questions with a binary response (yes/no). After asking about any impacts, respondents were asked about specific impacts on health, income and its sources, cost of food, food availability, travel, and agriculture activities (crop cultivation and harvest). Questions on sale of assets and livestock were used to assess negative coping strategies. These questions were only asked to the subset of households that reported any COVID-19 impact (Supplemental Table 3).

### Data Analysis

We reported the percentage of households experiencing food insecurity, perceived impacts of COVID-19 on livelihoods, and coping behavior at T1 and T2. Using data from both survey rounds, a food insecurity score was calculated by summing of positive responses to FIES questions to create a raw score from 0 to 8 for each household. For instance, a household with 0 score means that a nonpositive response was recorded for all 8 questions, whereas a score of 4 means that the household gave a positive response to any 4 out of 8 questions. Using these raw scores, we then determined percentage of households with any food insecurity (scores of 1-8), mild food insecurity (scores 1-3), moderate food insecurity (scores 4-6), and severe food insecurity (scores 7-8).^26,27^ We reported sample means for the proportion of households in each food insecurity category for both rounds (T1 and T2), then tested for a statistical difference in estimates between rounds using Pearson’s chi-square test.

For perceived COVID-19 impacts, we reported sample means for the percentage of households that experienced any COVID-19 impacts. Then, among households that experienced any COVID-19 impacts, we calculated sample means for households experiencing effects on health, income, cost of food, travel, food availability, agriculture activities (crop cultivation or harvest) and households that had to sell off assets or livestock to meet basic needs. We reported estimates for each round separately and then test for a statistical difference in estimates between rounds using Pearson’s chi-square test. Additionally, among households that indicated COVID-19 affected their income, we reported and tested for statistical differences between rounds for the effect of COVID-19 on sources of income (farming, fishing, nonfarm, wage labor, remittances, and benefits received in cash and kind).

Further, we conducted district-level analysis on reported sample means and tested for a difference between survey rounds for any reported food insecurity and any COVID-19 impacts. Among households that reported COVID-19 impacts, we also reported sample means at the district level for effects on health, income, food cost, food availability, agriculture activities, and sale of assets or livestock.

Further, using pooled data from both rounds, we tested the magnitude of association between any food insecurity (score of 1-8) and reported impact of COVID-19 on income using a binary logistic regression model. The model is specified below. Using the model, we reported adjusted odds ratios (aOR) with standard errors. As a robustness check, we test the coefficient on the COVID-19 variable using the Wald test.


$$FS_{i}=\alpha_{0}C_{i}+\alpha_{1}Z_{i}+\theta_{t}+\delta_{s}+{∊}_{i}$$

where, $fs_{i}$ is the household-level binary variable for food insecurity, *C_i_* is the reported COVID-19 household-level term, *Z_i_* is a vector of household-level variables at baseline (T1) (number of members, average number of assets owned, average land holding size, own house, and sex ratio), $\theta_{t}$ is the dummy for survey round, $\delta_{s}$ is the district-level factor variable, and ${∊}_{i}$ is the stochastic error term.

Stata 17 software was used for data cleaning and organization of the datasets.^28^ The descriptive means and regression analysis were conducted using RStudio.^29^ We only included households with complete data at both survey rounds, giving us an analytic sample of 1057 households.

## Results

### Sample Characteristics

The mean household size for the study sample was 4.3 members (Table 1). Most households lived in their own house (89%) and had an average agriculture landholding size of 1.2 hectares. The head of the household was, on average, 49 years of age, male (88%), and married (90%), and one-third had completed secondary school. Respondents were 46 years old on average, and two-thirds (64%) were male, married (87%), and had completed secondary school (40%). The respondent was the head of household in 70% of households, with spouse/partner of the head (25%) or son/daughter of the head (5%) being the other respondent types.

**Table 1.** Demographic Characteristics of Households That Participated in Both Rounds of Data Collection.^a^

	% or Mean (SD), (n = 1057)
Household characteristics	
Household size, No. of persons	4.3 (1.6)
Has a child ≤2 years	12
Has a member ≥60 years	31
Sex ratio categories	
Equal No. of male and female	31
More males	34
More females	35
Own house	89
Agriculture land holding size in hectares	1.2 (3.9)
Head of household characteristics	
Age, years	48.9 (12.2)
Male	88
Married	90
Education	
No school education	5
Primary, incomplete (grade 1-4)	19
Primary, complete (grade 5)	13
Secondary, incomplete (grade 6-10)	25
Secondary, complete (grade 10, O/L)	33
Higher secondary and above	5
Survey respondent characteristics	
Age, years	45.9 (12.6)
Male	64
Married	87
Education	
No school education	5
Primary, incomplete (grade 1-4)	16
Primary, complete (grade 5)	10
Secondary, incomplete (grade 6-10)	22
Secondary, complete (grade 10, O/L)	40
Higher secondary and above	7
Relationship with head of household	
Head of household	70
Spouse/partner of head	25
Son/daughter	5

Abbreviations: O/L, examination of General Certificate of Education Ordinary Level; SD, standard deviation.

^a^ Demographic data were collected from households only during the baseline survey round between December 2020 and February 2021.

### Food Insecurity Experience and Perceived COVID-19 Impacts

The percentage of households that reported any food insecurity in the last 30 days increased between survey rounds (T1: 75%, T2: 80%, *p* = .004; Table 2). While mild and moderate food insecurity increased by 5 percentage points (pp) ( *p* = .032) and 4 pp ( *p* = .030), respectively, severe food insecurity decreased by 4 pp ( *p* = .020). The proportion of households that perceived having been affected by COVID-19 or its mitigation measures was 84% in T1 and 89% in T2 ( *p* < .001). Compared to T1, among those who reported being affected by COVID-19, perceived impacts on agricultural activities were higher in T2 (T1: 64%, T2: 69%, *p* = .055) and perceived impacts on food availability were lower in T2 (T1: 71%, T2: 66%, *p* = .036). For other aspects affected by COVID-19, the change between rounds was not significant, with 75% or more reporting effects on income or jobs or livelihoods, food costs, and travel in both survey rounds. In terms of coping mechanisms, around half (55% in T1 and 56% in T1 and T2) of households reported having to sell assets or livestock to make ends meet.

**Table 2.** Food Insecurity Level and Perceived COVID-19 Impacts by Survey Round.

	T1 (%)	T2 (%)	*p* value^a^
Food insecurity in last 30 days	n = 1057	n = 1057	
Food insecurity level			
Any (≥1)	75	80	.004
Mild (1-3)	33	38	.032
Moderate (4-6)	26	30	.030
Severe (7-8)	16	12	.020
Perceived COVID-19 impact in last 6 months			
Any COVID-19 impact	84	89	<.001
Reported COVID-19 impact through:	n = 889	n = 994	
Poor health of household members	33	32	.490
Any income/job/livelihood loss	77	76	.580
Increased cost of food	84	83	.350
Not being able to travel	89	90	.230
Decreased food availability	71	66	.036
Loss of agricultural activities (crop cultivation or harvest)	64	69	.055
Sold livestock or assets	55	56	.590

^a^ Pearson’s chi-square test for difference between T1 and T2. T1: Baseline: December 2020-February 2021; T2: Follow-up: July-September 2021. Food insecurity and COVID-19 are reported measured at the household level. The food insecurity levels were created from the 8-item Food Insecurity Experience Scale (FIES), by classifying the raw score (0-8) into 4 different food insecurity levels: food secure (0), mildly food insecure (1-3), moderately food insecure (4-6), or severely food insecure (7-8).^26^

Using pooled data from both rounds, we found that 54% of households that reported COVID-19 affected their income were food insecure. Households reporting that COVID-19 affected their income were more likely to be food insecure compared to households reporting that COVID-19 did not affect their income (aOR: 2.56, *p* < .001, Supplemental Table 4).

### Impact of COVID-19 on Sources of Livelihood

While the overall percentage of households reporting negative impacts of COVID-19 on income was similar over time (T1: 77%, T2: 76%), impacts were more pervasive across income sources such as farming, livestock or poultry, fishing, and nonfarming at T2 (Table 3). For instance, at T2, 67% of households reported that their farming income was affected by COVID-19 compared to 56% in T1 ( *p* < .001). Similarly, COVID-19 impact on income from livestock/poultry and nonfarming sources was reported by 38% and 21% of households, respectively, in T2 compared to 27% and 17% of households in T1. In terms of support from government or other sources during COVID-19, 48% of households reported that income from Samurdhi (government cash support) was affected in T2, while 22% households reported effects on in-kind support such as food rations.

**Table 3.** Sources of Income Impacted by COVID-19 and Reasons for Crop and Harvest Impacts.

	T1 (%)	T2 (%)	*p* value^a^
Any income/job/livelihood loss^b^	77	76	.580
Among those who reported income impacts, sources of income impacted by COVID-19	n = 685	n = 717	
Farming	56	67	<.001
Livestock or poultry	27	38	<.001
Fishing	8	11	.066
Nonfarming	17	21	.026
Wage labor	37	33	.11
Remittances	4	5	.49
Samurdhi (cash) benefit	—	48	—
In-kind benefit (rations, etc)	—	22	—
Loss of agricultural activities (crop cultivation and harvest)^b^	64	69	.055
Among those who reported any cropping impact	n = 569	n = 647	
Both cultivation and harvest impact	12	18	<.001
Only crop cultivation impact	11	19	<.001
Only harvest impact	41	32	<.001

^a^ Pearson’s chi-square test for difference between survey rounds (T1 and T2). T1: Baseline: December 2020-February 2021; T2: Follow-up: July-September 2021. Questions on COVID-19’s impact on government’s cash transfer scheme “Samurdhi” and in-kind transfer were asked only during the follow-up round (T2).

^b^ Percentages for any income and any cropping impact are the same as the values reported in Table 2 and have been included here for clarity in terms of sample size for the results reported in this table.

Among the 64% of households in T1 and 69% of households in T2 reporting that COVID-19 affected their agricultural activities, both crop cultivation and harvest effects were reported by 12% of these households in T1 and 18% in T2 (* p* < .001; Table 3). Between T1 and T2, the percentage of households reporting that COVID-19 affected only their crop cultivation increased by 8 pp ( *p* < .001), while the percentage reporting COVID-19 effects on only harvest activities decreased by 8 pp ( *p* < .001).

Households also reported on how much COVID-19 increased or decreased each individual source of income (Supplemental Table 5). Among households that reported their farming income was affected, 73% reported a decline (small/medium/large/total loss) in T1 compared to 85% in T2. A similar reported fall in income was reported by households for fishing (T1: 70%, T2: 75%) and nonfarm income (T1: 85%, T2: 93%). Some households reported to have received cash benefits and other in-kind support during COVID-19. Approximately 81% of households reported an increase in the government cash (Samurdhi) transfer (for 60% of households it was a “small” increase), while 75% of households reported an increase in in-kind support (for 70% of households it was a “small” increase).

### District-Level Variation in Food Insecurity Experience and Perceived COVID-19 Impacts

**Figure 2.**
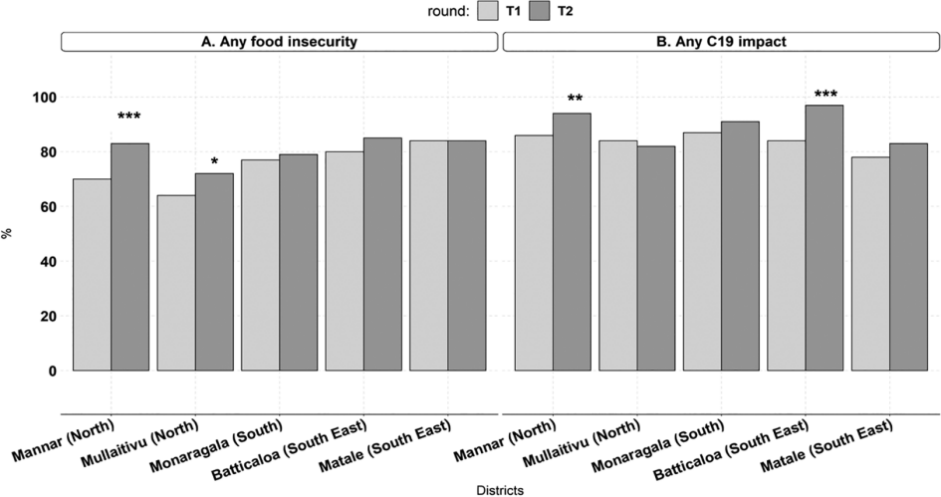
District-level food insecurity experience and perceived COVID-19 (C19) impacts by survey round.

There was variation in the percentage of food insecure households at the district level in both survey rounds (district range in T1: 64%-84%, T2: 72%-84%; Figure 2 Panel A). The percentage of food insecure households increased between T1 and T2 in the 2 northern districts, Mannar (T1: 70%, T2: 83%, *p* = .007) and Mullaitivu (T1: 64%, T2: 72%, *p* = .055), but stayed the same in the other 3 districts. Similar to food insecurity effects, the percentage of households reporting any perceived COVID-19 effects also varied at the district level (T1: 78%-87%, T2: 82%-97%; Figure 2 Panel B). Households experiencing any food insecurity increased between survey rounds in the northern district of Mannar (T1: 86%, T2: 94%, *p* = .037) and south-eastern district of Batticaloa (T1: 84%, T2: 97%, *p* < .001) but stayed the same in the other 3 districts.

Within districts, the proportion of households that perceived impacts of COVID-19 on health, income, food, travel, and having to sell assets or livestock were similar between T1 and T2, with a few exceptions (Supplemental Table 6). In Mullaitivu, the percentage of households that thought their income was affected by COVID-19 and/or the associated mitigation measures decreased by 9 pp (T1: 87%, T2: 78%, *p* = .022), and in Matale, there was a marginally significant decrease in the percentage of households who reported effects on food availability (T1: 67%, T2: 56%, *p* = .062). The percentage of households that suffered losses in agricultural activities increased over time in all districts (T1: 34%, T2: 53%, *p* < .001) except Batticaloa.

## Discussion

### Summary of Findings

Our analysis of 2 rounds of phone survey data fills a gap in the empirical literature on household experiences of food security and livelihood disruptions during COVID-19 in South Asia. Almost 3 quarters of households in rural Sri Lanka reported experiencing food insecurity and health or livelihood-related impacts due to COVID-19 or the associated mitigation measures. Despite a steep increase in the COVID-19 caseload over time and consistent mitigation measures of the government, the proportion of households that reported effects on income, cost of food, and travel was high in both survey rounds. Income sources such as farming, fishing, and livestock were perceived to be negatively affected during the pandemic. Some respite for households was reported from the cash and in-kind support received from the government and other agencies during COVID-19, though the increase was reported to be “small.” At the district level, food insecurity and perceived COVID-19 effects were pervasive and were the worst (among the 5 districts included in the study) in Mannar, Mullaitivu, and Batticaloa districts.

### Comparison With Other Studies and Interpretation of Our Findings

Our findings on high levels of food insecurity and negative perceived impacts of COVID-19 during 2 rounds of data collection during the pandemic in Sri Lanka are consistent with the literature on COVID-19 impacts in Africa and other South Asian countries.^30-34^ Other studies in Sri Lanka prior to COVID-19 show that agriculture and food systems were underdeveloped and vulnerable to shocks.^35,36^ An urban study found that compared to the prepandemic period in 2019, food insecurity increased during the pandemic.^37^

Our finding that households perceived a negative effect of COVID-19 on household income and employment aligns with another survey among low-income households in Sri Lanka.^38^ In their study, 60% to 80% of households reported reduced income in the pandemic period from April to Mar 2021. World Food Programme’s 2 rounds of surveys in rural Sri Lanka during COVID-19 also found that ∼70% of households experienced income loss and ∼55% of households reported using a coping mechanism during COVID-19, but WFP’s estimate of moderate and severe food insecurity, also measured using the FIES, was lower than our estimate (30% in September-October 2020 for the WFP study compared to 42% in our analysis).^39^ This difference in food insecurity estimate between the WFP and our study could be explained by differences in target population and method of analysis. While WFP’s study included rural, estate, and urban households, which could have potentially enrolled middle-class households, we used the program beneficiaries that were mostly low-income rural households. Additionally, while the WFP study used the Rasch model for analysis with adjusted severity parameters to the global standard that allows for cross-country comparison, we used the categorical food insecurity indicators that are used for more micro-level (individual or household) comparisons.^26,27^

We found that the percentage of households reporting that food availability was impacted by COVID-19 was lower in T2 than in T1 despite a higher COVID-19 caseload in T2 compared to T1. There could be several potential explanations for this finding. First, seasonal differences may be responsible. Our T2 survey was after the Maha or wet season in Sri Lanka, when paddy, the staple crop in the Sri Lankan diet, is cultivated. In an “above-normal” rainfall year, the production and harvest area of paddy during the wet season can be 50% higher compared to the Yala or dry season.^40^ In 2020, most parts of Sri Lanka received above-normal rainfall that resulted in an above-average harvest of paddy and other food crops in the Maha season.^41^ Second, several relief packages (cash and in-kind) and support programs were implemented as the pandemic unfolded, and rules for essential goods movement were relaxed. Agriculture production was supported by allowing normal farming activities and transport of farm inputs.^42^ During the first wave (October 2020-March 2021) when food prices in the retail market rose due to panic buying, the government initiated an online retailing platform to connect producers and buyers to ease food access.^43^ In 2020 lockdown period, the government also initiated a “Saubhagya” program to promote home gardens with the aim of utilizing unemployed labor and increase food availability.^42^ Our findings suggest that the benefits of the early relief packages were realized by the households in the period before T2 despite stricter policy measures and rising COVID-19 cases. The district-level variation in the food security could be explained by the prepandemic differences in household income. The income levels were relatively lower in the worst affected districts of Mannar, Mullaitivu, and Batticaloa districts.^44^ However, in the absence of data on income, food availability or substitution, and sociocultural difference during the pandemic, specific attribution is not possible.

The high prevalence of food insecurity reported by households in our study is an indication of fragile livelihoods in the rural parts of the country that may have been more exposed during the COVID-19 pandemic. Sustained levels of high food insecurity are associated with a range of negative health, nutrition, and well-being effects.^45-47^ Another concerning finding from our study was that half of the surveyed households reported that COVID-19 led them to sell assets or livestock to meet their basic needs, suggesting a need for short- and long-term support solutions to help mitigate the effects of shocks such as COVID-19. Lost or decreased income due to COVID-19, as seen in the preceding analysis, could have further economic consequences through reduced spending (potentially on nutrient-dense foods). This will further exacerbate malnutrition, especially given that, even before the pandemic, an estimated 53.5% of the Sri Lankan population could not afford a healthy diet.^48^

### Strengths and Limitations

To our knowledge, no other studies have reported food insecurity and perceived impacts of COVID-19 on health and livelihoods from rural Sri Lanka during the pandemic. Our data were from 5 districts covering various agroecological zones of the country and covered 2 waves of the pandemic that differed in terms of measures taken by the government to limit the spread of COVID-19. Although our study provides novel data, there are some limitations that need to be considered. First, all estimates are based on self-reported perceived impacts rather than objective measures. Second, we do not have prepandemic estimates of the FIES indicator for our sample. However, reports suggests that the country was highly vulnerable to shocks, and hunger was common even prior to the pandemic.^7,49^ Regardless, we cannot estimate the impact of COVID-19 on food insecurity using a pre/post-COVID-19 approach. Third, the survey data reported here are not nationally or district representative but cover an essential climate vulnerable demographic engaged in agriculture. Thus, the results should not be interpreted as being representative of the entire population of Sri Lanka. In future analyses, using an additional round of data (ie, T3, conducted in the same season as T1), we plan to assess impacts on household consumption and diets, that have been previously shown to be associated with food insecurity and worsened during the pandemic.^50^

## Conclusion

Our findings reinforce the need to build resilient food systems that can withstand shocks and structural changes that disrupt activities along the food value chain and lead to food insecurity.^51^ These disruptions can have long-term effects on poverty and hunger, economic inequalities, access to nutritious food, and health. Policymakers and international development agencies should identify vulnerable populations and help them prepare for future shocks through participation in inclusive, resilient, and nutrition-sensitive programs.

## Supplemental Material

Supplemental Material, sj-pdf-1-fnb-10.1177_03795721231197249 - Food Insecurity and Perceived Effects of COVID-19 on Livelihoods in Rural Sri Lanka Click here for additional data file. Supplemental Material, sj-pdf-1-fnb-10.1177_03795721231197249 for Food Insecurity and Perceived Effects of COVID-19 on Livelihoods in Rural Sri Lanka by Nishmeet Singh, Samuel Scott, Neha Kumar, Gayathri Ramani, Quinn Marshall, Kate Sinclair, Saman Kalupahana, Malika Fernando, Renuka Silva, Amila Perera, Renuka Jayatissa and Deanna Olney in Food and Nutrition Bulletin
