# Supplementary material for: Food Insecurity and Perceived Effects of COVID-19 on Livelihoods in Rural Sri Lanka
Source: Food Nutr Bull. 2023 Sep 13;44(4):229–39. doi: 10.1177/03795721231197249 (PMC10725086; doi:10.1177/03795721231197249)
Supplement: Supplemental Material, sj-pdf-1-fnb-10.1177_03795721231197249 - Food Insecurity and Perceived Effects of COVID-19 on Livelihoods in Rural Sri Lanka [file sj-pdf-1-fnb-10.1177_03795721231197249.pdf]

Food insecurity and perceived effects of COVID-19 on livelihoods in rural Sri Lanka

SUPPLEMENTAL MATERIALS ***(version: May 2023)***

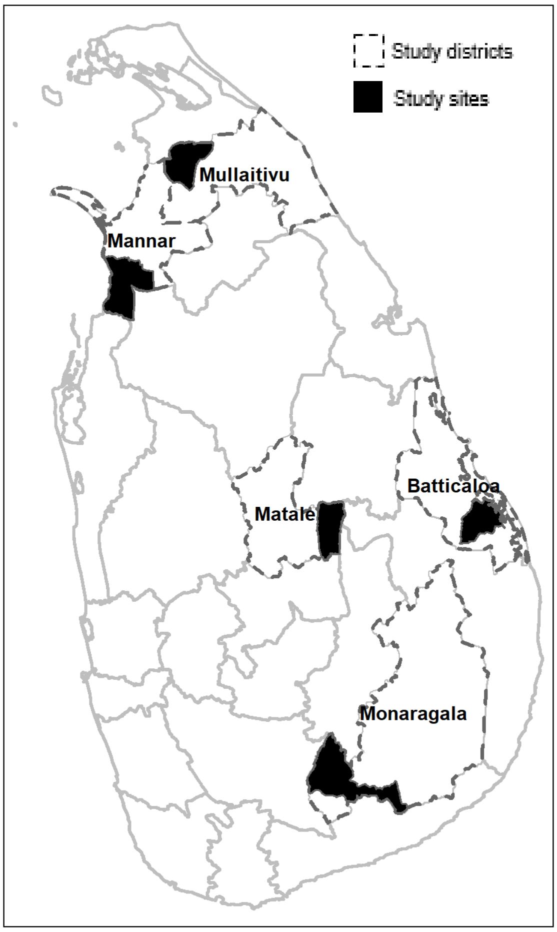

Supplemental Figure 1. Survey locations in Sri Lanka

Supplemental Table 1. Sample flow

	R5N (30 GNs)	Control (15 GNs)	Total Sample
**T1 (Baseline)**			
HHs in the electoral list	NA	5616	
		↓	
HHs for which phone numbers were obtained	NA	3265	
HHs randomly selected from list	NA	1400	
**HHs pre-contacted**	**1250**	**1400**	**2650**
HHs contacted by enumerator at baseline	1250	916	2166
Connected but refused/ not right person/ineligible	→184	→245	
Not connected	→207	→18	
**HHs consented for survey and follow-up**	**859**	**653**	**1512**
	↓	↓	↓
**Completed call 3 (survey modules: Assets; Knowledge; food insecurity; COVID impact; Livestock; Agriculture)***	**796**	**573**	**1369**
**T2 (follow-up)**			
HHs contacted	859	653	1512
	↓	↓	↓
HHs completed follow-up survey	638	487	1125
Missing responses for food security module	→6	→2	
Dropped for unavailability in both survey rounds	→27	→33	
**HHs selected for analysis (non-missing data in both rounds)**	**605**	**452**	**1057**

* Used for current analysis.

Notes: T1: (Baseline: December 2020-February 2021); T2: (Follow-up: July-September 2021). HHs-households; GNs- Grama Niladhari (smallest administrative unit);

Supplemental Table 2. List of survey modules by survey round and those included in the current study

Module label	Module Name	T1	T2	Included in the study
A1	Household Identification- Location	✔	✔	✔
A2	Household Roster	✔	✔	✔
A2B	Household Roster (New members)		✔	✔
B	Dwelling characteristics	✔		✔
C	Food consumption	✔		
D	Non-food expenditures	✔		
E	Nutrition Knowledge	✔	✔	
F	Exposure	✔	✔	
G	Household assets	✔		✔
H	Livestock ownership	✔		
I	Agriculture	✔		
J	Household food insecurity	✔	✔	✔
K	Coronavirus/COVID-19	✔	✔	✔
L	24 Hour Dietary Recall	✔	✔	
L2	Agriculture activities		✔	

Notes: T1: (Baseline: December 2020-February 2021); T2: (Follow-up: July-September 2021)

Supplemental Table 3. FIES and COVID-19 module used in both survey rounds

MODULE J: HOUSEHOLD FOOD INSECURITY		
**Say:** Now I would like to ask you some questions about food.		
**Q.no**	**Q. label**	**Response**
**J_1**	In the last 30 days, was there a time when you or others in your household worried about not having enough food to eat because of a lack of money or other resources?	1. Yes<br>0. No<br>98. Don't know
**J_2**	In the last 30 days, was there a time when you or others in your household were unable to eat healthy and nutritious food because of a lack of money or other resources?	1. Yes<br>0. No<br>98. Don't know
**J_3**	In the last 30 days, was there a time when you or others in your household ate only a few kinds of foods because of a lack of money or other resources?	1. Yes<br>0. No<br>98. Don't know
**J_4**	In the last 30 days, was there a time when you or others in your household had to skip a meal because there was not enough money or other resources to get food?	1. Yes<br>0. No<br>98. Don't know
**J_5**	In the last 30 days, was there a time when you or others in the household ate less than you thought you should because of a lack of money or other resources?	1. Yes<br>0. No<br>98. Don't know
**J_6**	In the last 30 days, was there a time when your household ran out of food because of a lack of money or other resources?	1. Yes<br>0. No<br>98. Don't know
**J_7**	In the last 30 days, was there a time when you or others in your household were hungry but did not eat because there was not enough money or other resources for food?	1. Yes<br>0. No<br>98. Don't know

**J_8**	In the last 30 days, was there a time when you or others in your household went without eating for a whole day because of a lack of money or other resources?	1. Yes<br>0. No<br>98. Don't know

MODULE K: CORONAVIRUS/COVID-19		
**K_ID**	Respondent ID	CAPI: Select name from drop-down menu
**Say:**<br>Now, I would like to ask you about Coronavirus/Covid-19 outbreak and how it has impacted your household.		
**K_1**	In the last 6 months, did the Coronavirus/Covid-19 outbreak or associated lockdowns impact your household in any way (for example livelihood, health, education, agriculture)?	Yes……………………………1<br>No……………………………..0 >> skip to next module
**K_2**<br>**List 1**	In the last 6 months, did the Coronavirus/Covid-19 outbreak or associated lockdowns impact your household through [List 1]?	
**A**	Increased cost of food	Yes……………………………1<br>No……………………………..0
**B**	Decreased availability of food	Yes……………………………1<br>No……………………………..0
**C**	Poor health of household members	Yes……………………………1<br>No……………………………..0
**D**	Not being to travel (i.e. to work, to the health center, to markets)	Yes……………………………1<br>No……………………………..0
**E**	Needing to sell livestock to pay for basic needs	Yes……………………………1<br>No……………………………..0
**F**	Needing to sell other assets to pay for basic needs	Yes……………………………1<br>No……………………………..0

**G**	Any loss of income/job/livelihood?		Yes……………………………1<br>No…………………………..0 >> if G is NO, skip to K_4
		**K_3**	**K_3_1**
		Was income from [List 2] affected?	How was the income affected?
	List 2	Yes……………………………1<br>No…………………………..0 >> skip to next item on List 2	Large increase…………………1<br>Medium increase……………….2<br>Small increase…………………3<br>Small decrease…………………4<br>Medium decrease………………5<br>Large decrease…………………6<br>Total loss………………………..7
**A**	Family farming		
**B**	Raising large livestock		
**C**	Raising small livestock		
**D**	Raising poultry		
**E**	Fishing		
**F**	Non-farm family business		
**G**	Wage employment of household members		
**H**	Remittances (abroad/within the country)		
**I**	Samurdhi or any other money transfers (i.e., Sri Lanka Military Assistance program) from government or non-governmental organizations		
**J**	Any in-kind transfers from governmental or non-governmental organizations (i.e., food vouchers)		
**K**	Other, specify		
**K_4**	In the last 6 months of the Coronavirus/Covid-19 outbreak, were you able to perform the normal crop		Yes……………………………1 >> skip to K_6

	cultivation activities like preparing your land, purchasing inputs, planting, weeding, planning the harvest?	No……………………………..0
**K_5**	What are the main reasons you were not able to perform the normal crop cultivation activities?<br><br>H: Enumerator do not read options out loud<br><br>H: Select all that apply	Advised to stay home ....1<br>Reduced availability of hired labor ....2<br>Restrictions on movement /travel ....,3<br>Unable to purchase inputs ....4<br>Higher input prices……….…..5<br>Unable to sell / transport outputs ….................................6<br>Need to care for ill family member ....7<br>Weather related problems…......8<br>Lack of inputs…………………9<br>Other, specify 96
**K_6**	In the last 6 months of the Coronavirus/Covid-19 outbreak, did you face any difficulties in selling harvested agriculture produce due to the lockdown related to Coronavirus/Covid-19?	Yes……………………………..1<br>No……………………………..0 >> skip to next module
**K_7**	What kind of difficulties did you face in selling harvested agriculture produce due to the lockdown related to Coronavirus/Covid-19?<br><br>H: Enumerator do not read options out loud<br><br>H: Select all that apply	Poor demand/no buyers………..1<br>Markets were closed…………...2<br>Restrictions on movement /travel ………………………….3<br>No means of transportation to markets…………………..….4<br>Storage facilities were closed /inaccesible……………………..5<br>Lower prices for agri produce….6<br>Delayed payments for the produce ………………………...7<br>Not yet harvested……………….8<br>Other, specify…………………..96

Supplemental Table 4. Regression results of association between any food insecurity and perceived COVID-19 income impacts

	Dependent variable: **Any food insecurity**
	**Adjusted Odds Ratio (SE)**
**COVID-19-associated income impact**	2.556*** (0.308)
**Covariates:**	
Number of household members	1.078* (0.044)
Number of assets owned by the household	0.894***(0.012)
Agriculture land holding (hectares)	0.963***(0.014)
Own house:	0.931 (0.177)
Sex ratio:	
More males or only males	0.677*** (0.099)
More females or only females	0.730**(0.109)
District:	
Mannar	0.722 (0.151)
Matale	2.155***(0.469)
Monaragala	1.277 (0.240)
Mullaitivu	0.661** (0.119)
**Round: T2**	**1.314** (0.151)**
Constant	16.829***(6.248)
Observations	1,956

***Significant at the 1 percent level.

**Significant at the 5 percent level.

*Significant at the 10 percent level.

Notes: SE: Standard Error

Supplemental Table 5. Reported degree of COVID-19's effect on sources of income

	T1	T2
**Farming**		
Increase (large/med/small)	27%	15%
Decrease (large/med/small)	66%	81%
Total loss	7%	4%
**Fishing**		
Increase (large/med/small)	30%	28%
Decrease (large/med/small)	51%	59%
Total loss	19%	13%
**Non-farm income**		
Increase (large/med/small)	15%	7%
Decrease (large/med/small)	72%	70%
Total loss	13%	23%
**Wage income**		
Increase (large/med/small)	30%	9%
Decrease (large/med/small)	51%	64%
Total loss	19%	27%
**Remittance income**		
Increase (large/med/small)	40%	25%
Decrease (large/med/small)	37%	53%
Total loss	23%	22%
**Samurdhi**		
Large increase	-	0%
Medium increase	-	21%
Small increase	-	60%
Small decrease	-	4%
Medium decrease	-	7%
Large decrease	-	5%
Total loss	-	3%
**In-kind**		
Large increase	-	3%
Medium increase	-	14%
Small increase	-	69%
Small decrease	-	45%
Medium decrease	-	1%
Large decrease	-	3%
Total loss	-	5%

Notes: T1: (Baseline: December 2020-February 2021); T2: (Follow-up: July-September 2021)

Supplemental Table 6. **Food insecurity experience and perceived COVID-19 impacts by sample districts and survey round**

	**Mannar (North)** N=155			**Mullaitivu (North)** N=248			**Monaragala (South)** N=252			**Batticaloa (South East)** N=222			**Matale (South East)** N=180		
	T1	T2	p-value	T1	T2	p-value	T1	T2	p-value	T1	T2	p-value	T1	T2	p-value
	%	%		%	%		%	%		%	%		%	%	
Any food insecurity	70	83	0.007	64	72	0.055	77	79	0.590	80	85	0.170	84	84	0.890
Any COVID-19 impact	86	94	0.037	84	82	0.630	87	91	0.120	84	97	<0.001	78	83	0.230
**Reported COVID-19 impact through:**															
Health of household members	35	27	0.140	34	29	0.300	27	30	0.410	35	38	0.540	38	33	0.380
Any income/job/livelihood loss	89	82	0.110	87	78	0.022	58	67	0.077	91	87	0.170	62	67	0.450
Increased cost of food	87	86	0.930	79	73	0.110	86	85	0.850	83	83	0.960	87	87	0.980
Decreased food availability	84	79	0.360	69	65	0.330	60	55	0.240	80	79	0.880	67	56	0.062
Loss of agriculture activities	61	62	0.840	59	67	0.10	78	84	0.069	34	53	<0.001	60	52	0.190
Sold livestock or assets	67	75	0.140	59	54	0.330	43	37	0.170	60	67	0.140	47	53	0.320

Notes: T1: Notes: T1: (Baseline: December 2020-February 2021); T2: (Follow-up: July-September 2021). p-values are reported using Pearson's Chi-squared test between T1 and T2
